# Clinical and economic impact of antibiotic resistance in developing countries: A systematic review and meta-analysis

**DOI:** 10.1371/journal.pone.0189621

**Published:** 2017-12-21

**Authors:** Raspail Carrel Founou, Luria Leslie Founou, Sabiha Yusuf Essack

**Affiliations:** 1 Antimicrobial Research Unit, School of Health Sciences, University of KwaZulu-Natal, Durban, South Africa; 2 Department of Clinical Microbiology, Centre of Expertise and Biological Diagnostic of Cameroon, Yaoundé, Cameroon; 3 Department of Food Safety and Environmental Microbiology, Centre of Expertise and Biological Diagnostic of Cameroon, Yaoundé, Cameroon; Ross University School of Veterinary Medicine, SAINT KITTS AND NEVIS

## Abstract

**Introduction:**

Despite evidence of the high prevalence of antibiotic resistant infections in developing countries, studies on the clinical and economic impact of antibiotic resistance (ABR) to inform interventions to contain its emergence and spread are limited. The aim of this study was to analyze the published literature on the clinical and economic implications of ABR in developing countries.

**Methods:**

A systematic search was carried out in Medline via PubMed and Web of Sciences and included studies published from January 01, 2000 to December 09, 2016. All papers were considered and a quality assessment was performed using the Newcastle-Ottawa quality assessment scale (NOS).

**Results:**

Of 27 033 papers identified, 40 studies met the strict inclusion and exclusion criteria and were finally included in the qualitative and quantitative analysis. Mortality was associated with resistant bacteria, and statistical significance was evident with an odds ratio (OR) 2.828 (95%CI, 2.231–3.584; p = 0.000). ESKAPE pathogens was associated with the highest risk of mortality and with high statistical significance (OR 3.217; 95%CIs; 2.395–4.321; p = 0.001). Eight studies showed that ABR, and especially antibiotic-resistant ESKAPE bacteria significantly increased health care costs.

**Conclusion:**

ABR is associated with a high mortality risk and increased economic costs with ESKAPE pathogens implicated as the main cause of increased mortality. Patients with non-communicable disease co-morbidities were identified as high-risk populations.

## Introduction

Antimicrobial resistance (AMR) is the ability of bacteria, parasites, viruses and fungi to grow and spread in the presence of antimicrobial medicines that are normally active against them. AMR occurs via a range of resistance mechanisms, such as a modified antimicrobial target, enzymatic hydrolysis/degradation, efflux and impermeability. This resistance is mediated by diverse resistance genes that evolve as a result of antimicrobial selection pressure exerted by the appropriate and/or inappropriate use of antimicrobial medicines, and is aggravated by the void of new antimicrobial agents in the current therapeutic pipeline [1, 2]. AMR increases health-care costs, length of stay in hospitals, morbidity and mortality in both developed and developing countries [3]. A recent report estimated that 10 million deaths will be attributed to AMR by 2050, and 100 trillion USD of the world’s economic outputs will be lost if substantive efforts are not made to contain this threat [1, 4, 5].

The World Health Organization (WHO) published the first global surveillance report on antibiotic resistance (ABR) in 2014 to show the clinical impact of resistant bacteria in WHO regions across the world. This reported shown that five out of the six WHO regions had more than 50% resistance to third generation cephalosporins and fluoroquinolones in *Escherichia coli* and methicillin resistance in *Staphylococcus aureus* in hospital settings. Similarly, more than 50% resistance to third generation cephalosporins and carbapenems was reported in *Klebsiella pneumoniae*. The report attributed 45% of deaths in both Africa and South-East Asia to multi-drug resistant (MDR) bacteria. It further revealed that *K*. *pneumonia*e resistant to third generation cephalosporins was associated with elevated deaths in Africa (77%), the Eastern Mediterranean region (50%), South East Asia (81%) and Western Pacific region (72%) [2].

Several resistant bacteria have been increasingly involved in infectious diseases in humans, specifically, *Enterococcus spp*, *S*. *aureus*, *K*. *pneumoniae*, *Acinetobacter baumannii*, *Pseudomonas aeruginosa* and *Enterobacter spp*. They are collectively termed ESKAPE and recently gained further global attention by being listed by the WHO as priority antibiotic-resistant bacteria to guide research, discovery, and development of new antibiotics [5]. The particularity of these bacteria is their ability to develop high level resistance to multiple drugs, thereby limiting therapeutic options and increasing morbidity and mortality. Numerous studies have confirmed that ESKAPE bacteria and their resistant clones, are actively transmitted in hospitals and communities in both developed and developing countries. The threat posed by these resistant bacteria is however exacerbated in developing countries due to sub-optimal hygiene conditions, poor infection, prevention and control measures, lack of surveillance and the dearth antimicrobial stewardship programs [6, 7]. Reports have shown high isolation rates of methicillin resistant *S*. *aureus* (MRSA) in healthcare settings in Cameroon (72%), South Africa (52%), Ethiopia (42.8%), Nigeria (29.6%), Kenya (27.7%), Ivory Cost (16.8%) and Morocco (14.4%) [2, 8–10]. In 2008, the prevalence of nosocomial acquired and MDR infections due to Enterobacteriaceae isolated from blood cultures were 57.1% and 15.4% respectively, in South Africa [11]. Likewise, rapid increases in the rates of infections due to carbapenemase-producing *K*. *pneumonia*, metallo-beta-lactamase-producing *A*. *baumannii* (MBL-AB), metallo-beta-lactamase-producing *P*. *aeruginosa* (MBL-PA), and extended-spectrum beta-lactamase (ESBL) producing *Enterobacter spp*. have been reported across the world [12–14]. In Saudi Arabia, the rate of *P*. *aeruginosa* producing carbapenemase was 33%, of which 27% were MBL-producers [15], while in India, a 22.4% prevalence of *P*. *aeruginosa* producing MBLs was reported in tertiary care hospitals [16].

MDR-ESKAPE bacteria have been reported in hospital acquired infections (HAI), particularly in intensive care units (ICUs) where immune-compromised patients suffering from some non-communicable diseases (NCDs) including diabetes, cancers, chronic lung, cardiovascular and kidney diseases were highly affected [6, 17–22]. The emergence and spread of these highly resistant bacteria in hospital care settings could thus have negative health repercussions and be an obstacle for the treatment of infections of patients with these NCDs [18, 23].

Despite the evidenced threat posed by ABR, information on its clinical and economic impact is limited in developing countries, and thus impede appropriate interventions for its containment [24, 25]. Heightened awareness of policy-makers, health care workers, and the general population about the risks associated with ABR is essential to preserve antibiotics for future generations [26, 27]. Hence, the aim of this study was to analyze the published literature on the clinical and economic impact of ABR in developing countries, in order to inform containment strategies such as antimicrobial stewardship programs and infection prevention and control measures in these nations.

## Methods

The Preferred Reporting Items for Systematic Reviews and Meta-analyses (PRISMA) and Meta-analysis of Observational Studies in Epidemiology (MOOSE) guidelines were followed [28, 29].

### Ethical consideration

This systematic review and meta-analysis was based on published reports, and was therefore exempt from ethical approval.

### Systematic review of the literature

A systematic search was carried out independently by RF and LF, in Medline via PubMed and Web of Sciences from January 2000 to December 09, 2016, using a combination of boolean operators (AND/OR), Medical Subject Heading (MeSH) and pre-defined keywords. Only published after 2000 were considered to ensure that the analysis focuses on contextual literature that depict current resistance patterns, infection rates, prevention measures, and clinical practice guidelines. Peer-reviewed papers in English and French on the clinical and/or economic impacts of ABR in developing countries were retrieved and independently evaluated for eligibility by RF and LF based on titles and abstracts (Table 1). Thereafter, the full-texts of eligible papers were assessed according to pre-defined inclusion and exclusion criteria (Table 1), with inconsistencies and disagreements being resolved by consensus. Efforts were made to contact the authors when data was missing and full-texts could not be retrieved, and a hand search was conducted in the reference list of all selected papers.

**Table 1:** Eligibility criteria.

***Inclusion criteria***
- Original research - Minimum of 20 patients - Studies conducted in developing countries as defined by World Bank criteria - Report on association between resistant bacteria and clinical outcome and/or financial impact - Antimicrobial susceptibility testing done by either disk diffusion, broth micro-dilution, agar dilution, E-test or VITEK using - CLSI/EUCAST/SFM guidelines - Papers published in French and English - Studies published from January 1, 2000
***Exclusion criteria***
- Reports of antibiotic resistance unrelated to clinical outcome nor economic impact - Reports on parasites, viruses and fungi - Reports on treatment comparisons - Studies conducted in developed countries as defined by World Bank criteria - Reports published in languages other than French and English - Antibiotic resistance in wildlife, companion and aquatic animals - Grey literature, conference abstracts, reviews, meta-analysis, letters to editor, correspondence, editorials, comments and case reports. - Studies published before January 1, 2000

### Screening and data extraction process

Papers were managed using EndNote (version X7.7.1, Thomson Reuters) and the data from eligible papers was abstracted independently by two authors (RF and LF) using a standardized data extraction spreadsheet in Excel^®^ (Microsoft^®^ Office Excel 2016). Relevant data from papers included countries, WHO regions, World Bank classification, publication year, type of study, participant characteristics (number of participant, diseases, age), hospital’ ward, bacteria, follow-up period, length of stay in hospital, mortality related to resistant bacteria, and, costs as described in Table 2.

**Table 2:** Description of eligible papers included in the systematic review.

Country	Year	Type of study	Study population	Infection type	Hospital’ ward	Bacteria	Sample size cases/ controls	Length of stay^2^ (%)		Mortality^3^ n/N (%)		References
								Case group	Control group	Case group	Control group	
**STUDIES REPORTING IMPACT OF ABR ON THE MORBIDITY ONLY**												
Turkey	2015	Retrospective cohort	NR	Nosocomial BSI	ICU	*A*. *baumannii*	41/45	25.49 days (%NR)	22.80 days (%NR)	NR	NR	[3]
Turkey	2008	Prospective case—control	Adults>16 years old	Nosocomial Infections	ICU and others	*A*. *baumannii*	66/57	20.8 days (65.2%)	15.4 days (40.4%)	NR	NR	[30]
**STUDIES REPORTING IMPACT OF ABR ON THE MORTALITY ONLY**												
Brazil	2009	Retrospective case-control	Adults >14 years old	Nosocomial infections	Medical-surgical ICU	*P*. *aeruginosa*	63/182	NR	NR	31/63 (49%)	61/182 (33%)	[31]
Brazil	2009	Case-control	Adults > 18 years old	BSI	NR	*E*. *coli* and *K*. *pneumoniae*	30/64	NR	NR	7/30 (23.3%)	12/64 (18.8%)	[32]
China	2004	Case-control and Retrospective cohort	All ages	MDR-HAI	Various wards^1^	*P*. *aeruginosa*	44/68	NR	NR	24/44 (54.5%)	11/68 (16.2%)	[33]
China	2012	Retrospective	Children < 15 years old	Pneumonia	Pediatric ICU	*A*. *baumannii*	115/45	NR	NR	21/115 (18.26%)	2/45 (4.44%)	[34]
China	2015	Retrospective Case-Control	NR	MRSA infections	Various	*S*. *aureus*	57/116	NR	NR	12/57 (21%)	9/116 (8%)	[35]
Colombia	2014	Case-Control	All ages	CR-KP Infection	ICU	*K*. *pneumoniae*	61/122	NR	NR	31/61 (50.8%)	25/122 (20.4%)	[36]
India	2014	NR	Neonates	BSI	Neonatal ICU	*A*. *baumannii*	33/32	NR	NR	9/33 (27.3%)	3/32 (9.4%)	[37]
Malaysia	2009	Case-control	NR	Nosocomial AB BSI	NR	*A*. *baumannii*	53/56	NR	NR	25/53 (47.2%)	14/56 (25%)	[38]
Malaysia	2011	Cross-sectional descriptive and case-control	NR	IR-*A*. *baumannii* BSI	NR	*A*. *baumannii*	15/41	NR	NR	9/15 (64.3%)	15/41 (40.5%)	[39]
Mexico	2000	Case-control	Children	Pneumoniae	NR	*S*. *pneumoniae*	25/24	NR	NR	11/25 (44%)	7/24 (29%)	[40]
Thailand	2011	Case-control	Adults >15 years old	MDR-*A*. *baumannii* bacteremia	In and out-patient departments	*A*. *baumannii*	24/25	NR	NR	22/24 (91.7%)	12/25 (48%)	[41]
Thailand	2012	Case-control	Adults >15 years old	ESBL-producing bacteria in septicemia	In and out-patient departments	*E*. *coli*	32/113	NR	NR	9/32 (29%)	13/113 (11.5%)	[42]
Thailand	2015	Case-control	Adults>18 years old	HAI	ICU and general wards	*A*. *baumannii*	139/132	NR	NR	79/139 (57%)	3/132 (2%)	[43]
Thailand	2015	Retrospective cohort	Adults	Ventilator Associated Pneumoniae	ICU	*A*. *baumannii*	220/33	NR	NR	125/220 (56.8%)	7/33 (21.2%)	[44]
**STUDIES REPORTING IMPACT OF ABR ON THE MORBIDITY AND MORTALITY**												
Brazil	2015	Case-control	Cancer children <18 years old	MDR-GNB Infection	Oncology pediatric ICU	Gram Negative Bacteria	47/54	8 days (63.8%)	2 days (37%)	12/47 (25.5%)	9/54 (16.7%)	[17]
Brazil	2006	Retrospective cohort	>1-year-old	BSI	Various wards^1^	*S*. *aureus*	61/50	>10 days (65.9%)	>10 days (34.1%)	33/61 (54.9%)	12/50 (24.7%)	[45]
Brazil	2006	Retrospective cohort	All ages	BSI	Various wards^1^	*K*. *pneumoniae*	56/52	>10 days (56.2%)	>10 days (43.8%)	18/56 (69.2%)	8/52 (30.8%)	[46]
Brazil	2008	Case-control	Adults	VAP	ICU	*S*. *aureus*	29/32	>8 days (89.7%)	>8 days (90.6%)	11/29 (37.9%)	8/32 (25%)	[47]
Brazil	2012	Case-control	Adults > 18 years old	Bacteremia	ICU	*P*. *aeruginosa*	29/48	43 days (NR)	43.1 days (NR)	13/29 (44.8%)	26/48 (54.2%)	[22]
China	2012	Retrospective cohort	> 1 year old	BSI	Various wards^1^	*S*. *aureus*	75/43	55.3 days (NR)	38.7 days (NR)	25/75 (33.3%)	8/43 (18.6%)	[48]
China	2015	Retrospective	Geriatric inpatients	Bacteremia	Various wards^1^	*A*. *baumannii*	39/86	36.7 days (NR)	36.1 days (NR)	31/39 (79.5%)	38/86 (44.2%)	[49]
China	2015	Retrospective case-control	NR	Enterococci infections	Various wards^1^	*Enterococci*	44/176	37 days (NR)	17 days (NR)	3/44 (6.8%)	3/176 (1.7%)	[50]
Colombia	2014	Prospective cohort	Adult	CR-*A*. *baumannii* Infections	ICU	*A*. *baumannii*	104/61	19 days (NR)	16.2 days (NR)	42/104 (40%)	13/61 (21%)	[51]
India	2014	Observational	Adults	Septicemia	Various wards	GNB and GPB	133/87	14 days (NR)	11 days (NR)	16/133 (12%)	2/87 (2%)	[52]
Jordan	2015	Matched case-control	Cancer patients	Nosocomial *A*. *baumannii* infections	Medical-surgical ICU	*A*. *baumannii*	161/262	12 days (NR)	3 days (NR)	118/161 (73.3%)	142/232 (61.2%)	[53]
Palestine	2009	Prospective case—control	Neonates	Nosocomial septicemia	Neonatal ICU	*A*. *baumannii*	40/100	20 days (62.5%)	20 days (35%)	15/40 (37.5%)	12/100 (13.2%)	[54]
Senegal	2016	Classic retrospective cohort and retrospective parallel cohort	All ages	ESBL- producing Enterobacteriaceae	Various wards^1^	*K*. *pneumoniae* *Enterobacter* *E*. *coli*	110/76	22.6 days (NR)	14 days (NR)	52/110 (47.3%)	17/76 (22.4%)	[55]
Thailand	2007	Prospective case—control	Adults	HAI	Various wards^1^	*E*. *coli* and *K*. *pneumoniae*	74/74	22.5 days (NR)	17.5 days (NR)	26/74 (35.1%)	12/74 (16.2%)	[56]
Thailand	2008	Cohort	Adults	Community-onset BSI	Various wards^1^	*E*. *coli* and *K*. *pneumoniae*	36/108	8 days (NR)	6 days (NR)	13/36 (36%)	16/108 (15%)	[57]
Thailand	2014	Retrospective cohort	Adults>18 years old	HAI	Various wards^1^	*A*. *nosocomialis* and *A*. *pittii*	25/58	9 days (NR)	4 days (NR)	3/25 (12%)	20/58 (35%)	[58]
Thailand	2009	Retrospective cohort	Adult> 15 years old	Nosocomial BSI	Various wards^1^	*A*. *baumannii*	67/131	37 days (NR)	27 days (NR)	35/67 (52.2%)	26/131 (19.9%)	[59]
Thailand	2006	Cross-sectional	All ages	Community-acquired pneumoniae	NR	*S*. *pneumoniae*	22/42	12.2 days (NR)	15.5 days (NR)	2/22 (9.1%)	4/42 (9.5%)	[60]
Thailand	2009	Case-control	Adult>18 years old	Nosocomial BSI	Various wards^1^	*E*. *coli* and *K*. *pneumoniae*	51/94	26 days (NR)	16 days (NR)	26/51 (51.0%)	28/94 (29.8%)	[61]
Thailand	2013	Retrospective Case-control	Neonates	CR- *A*. *baumannii* Bacteremia	Neonatal ICU	*A*. *baumannii*	14/44	34 days (NR)	24.5 days (NR)	6/14 (42.9%)	3/44 (5.9%)	[62]
Thailand	2016	Retrospective Case-control	Neonates	VAP	Neonatal ICU	*A*. *baumannii*	63/25	51 days (NR)	41 days (NR)	10/63 (15.9%)	0/25 (0%)	[19]
Turkey	2015	Observational retrospective cohort	All ages	HAI	ICU	*K*. *pneumoniae*	47/51	19 days (37.3%)	11 days (29.94%)	21/47 (44.7%)	26/51 (51%)	[63]
Turkey	2000	Retrospective	Adults	Bacteremia	ICU	*S*. *aureus*	46/55	50.3 days (NR)	32.7 days (NR)	15/46 (32.6%)	7/55 (12.7%)	[21]
Turkey	2015	NR	NR	Nosocomial infections	Emergency ICU and Pediatric ICU	*P*. *aeruginosa*	32/8	20.58 days (NR)	6.33 days (NR)	14/32 (43.8%)	2/8 (25%)	[64]

LOS: Length of stay; NR: Not reported; BSI: Bloodstream infection, HAI: Hospital-acquired infection, VAP: Ventilator-Associated Pneumoniae; CR: Carbapenem-resistant; GNB: Gram negative bacteria; GPB: Gram positive bacteria

1 various wards

2 LOS attributed to the specific bacteria responsible of the infections

3: Overall mortality attributed to the specific bacteria responsible of the infections, ICU: Intensive Care Unit.

### Statistical analysis

Meta-analyses were undertaken using Comprehensive Meta-analysis software (Biostat, Inc., New Jersey, USA) version 3 for Windows, to determine overall mortality risk associated with resistance. Sub-group analyses for mortality were conducted for the data by WHO region, World Bank classification, countries, group of bacteria, and bacterial species where there were three or more studies that could be combined. Forest plots were performed to assess the significance of the results and generated using 95% confidence intervals (CIs). Analyses were undertaken across sub-groups for the selected outcome and the results presented as odds ratios. Studies were weighted in favor of those with narrower confidence intervals (more precise results), and the random-effects method was used to provide more confident data considering heterogeneity within and between reports. The *I-square (I*^*2*^*)* statistic with cut-off values of 25, 50 and 75% was used to assess low, moderate and high heterogeneity respectively, and a *p-*value of <0.05 was considered statistically significant. Publication bias was evaluated using the funnel plot and statistical egger’s test.

### Quality assessment

Quality assessment was performed independently by RF and LF using the Newcastle-Ottawa quality assessment scale (NOS) for each study included in the systematic review and meta-analysis [65]. NOS assesses methodological quality, based on three-dimensional criteria and included (i) selected population, (ii) comparability of groups, and (iii) outcome/exposure of interest. Studies were scored using a scale with a possible maximum of eight points where a score ≥ 6 indicated high-quality studies, a score between 3–6 as moderate and a score ≤ 3 as low quality.

## Results

### Literature search and study selection

**Fig 1:**
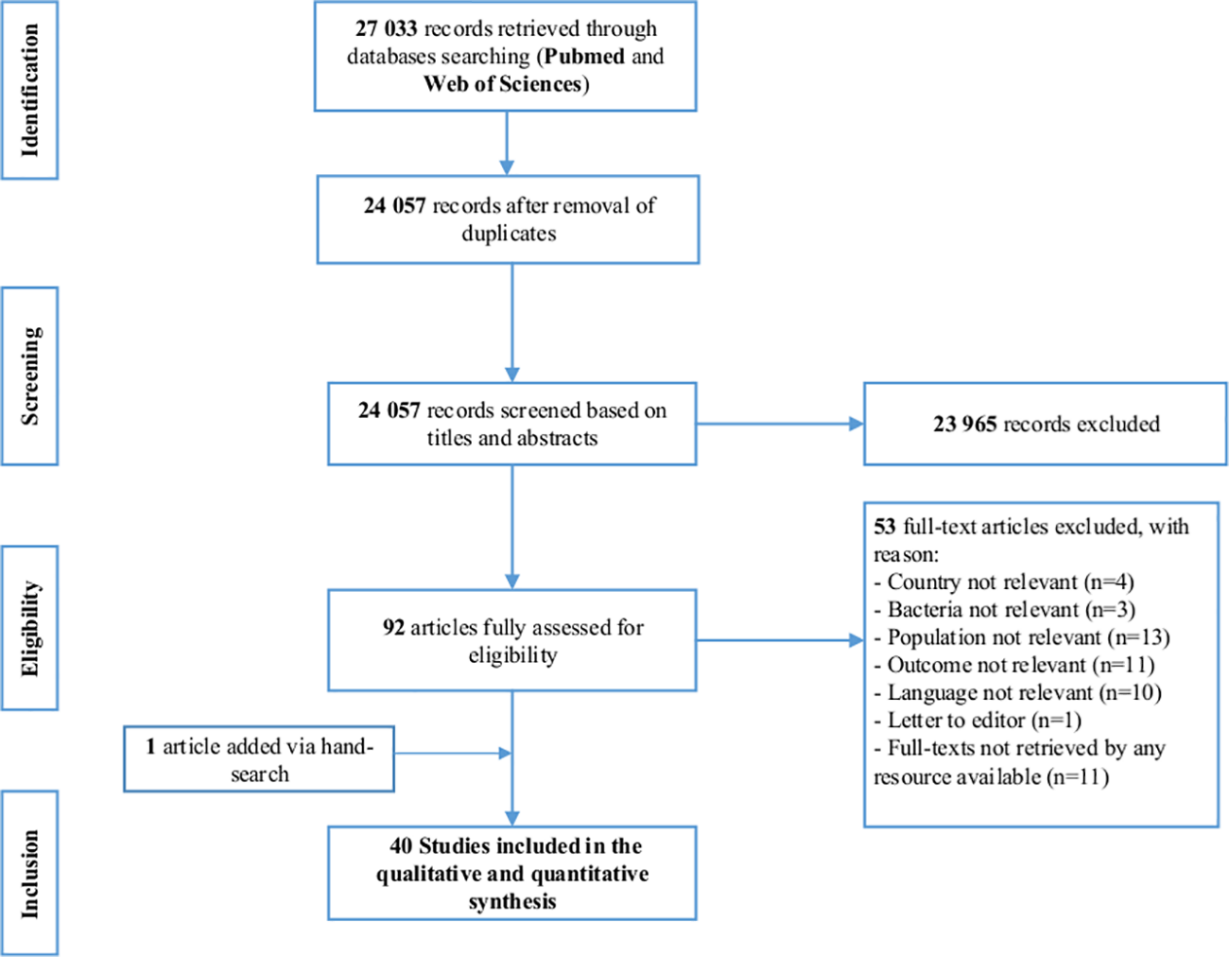
Prisma Flow-chart illustrating the study selection process.

The systematic search conducted in the two electronic databases generated 27 033 papers. A total of 24 057 papers were screened for probable inclusion according to titles and abstracts after de-duplication. Of these, the full texts of 92 eligible papers were fully evaluated based on predefined inclusion and exclusion criteria. One article was added following a hand-search in the reference lists of included papers. Forty studies were finally eligible for the qualitative and quantitative analysis (Fig 1), of which 18 were of high quality, while 15 and seven were moderate and low quality respectively.

### Description and characteristics of studies included in systematic review

**Fig 2:**
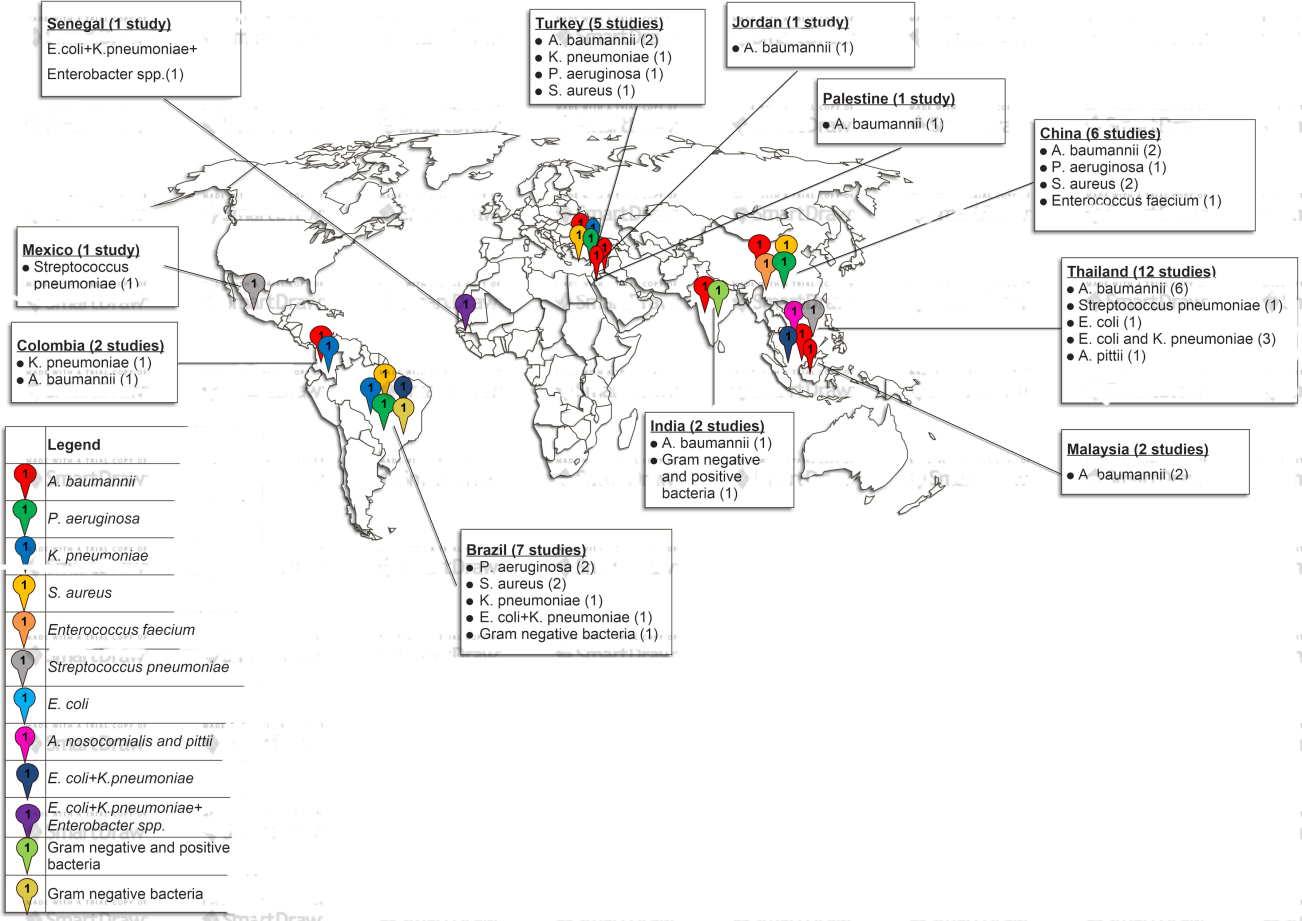
Graphical representation of AMR in developing countries included in the study.

The majority of data analyzed were obtained from single center studies conducted in 11 countries. Thirty percent (n = 12) of the observational studies on ABR were conducted in hospitals and communities in Thailand, the rest were performed in 10 different-countries namely Brazil (n = 7; 17.5%), China (n = 6; 15%), Turkey (n = 5; 12.5%), Colombia (n = 2; 5%), Malaysia (n = 2; 5%), India (n = 2; 5%), Mexico (n = 1; 2.5%), Jordan (n = 1; 2.5%), Palestine (n = 1; 2.5%), and Senegal (n = 1; 2.5%) (Table 2 and Fig 2).

Fourteen studies investigated the impact of ABR on mortality, two reported its impact on morbidity only (Table 2) while 24 considered both morbidity and mortality concomitantly. Eight studies reported on the economic consequences of ABR (Table 3). *A*. *baumannii* (n = 14; 35%), *K*. *pneumoniae* (n = 6; 15%), *S*. *aureus* (n = 5; 12.5%), *P*. *aeruginosa* (n = 4; 10%) represented the main pathogens reported with ICUs being the principal hospital ward concerned (Tables 2 and 3).

**Table 3:** Studies describing mortality rate associated with resistant and MDR ESKAPE bacteria.

Authors	Hospital Wards	Bacteria	Mortality rate	P-value	References
Al Jarousha et al. (2009)	Neonatal ICU	MDR-*A*. *baumannii* (15/40)	37.5%	0.001	[54]
		Susceptible *A*. *baumannii* (12/100)	12%		
Anunnatsiri et al. (2011)	ICU	MDR-*A*. *baumannii* (22/24)	91.7%	0.001	[41]
		Susceptible *A*. *baumannii* (12/25)	48%		
Amer et al. (2015)	Emergency ICU /Pediatric ICU	CR-MBLP-*P*. *aeruginosa* (14/32)	43,8%	0.2	[64]
		CR-MBLN-*P*. *aeruginosa* (2/8)	25%		
Furtado et al. (2009)	ICU	Imipenem-resistant *P*. *aeruginosa* (31/63)	49%	0.02	[31]
		Imipenem-susceptible *P*. *aeruginosa* (61/182)	33%		
Marra et al. (2006)	ICU	ESBL-producing *K*. *pneumoniae* (18/56)	32.14%	0.042	[46]
		Non-ESBL *K*. *pneumoniae* (8/52)	15.38%		
Moreira et al. (2008)	ICU	ORSA (11/29)	37.9%	0.41	[47]
		OSSA (8/32)	25%		
Serefhanoglu et al. (2009)	ICU	MDR-ESBL-producing-*E*. *coli* and *K*. *pneumoniae* (7/30)	23.3%	0.606	[32]
		Non-MDR-ESBL-producing-*E*. *coli* and *K*. *pneumoniae* (12/64)	18.8%		
Tuon et al. (2012)	ICU	Carbapenem-resistant *P*. *aeruginosa* (13/29)	54.2%	0.043	[22]
		Carbapenem-susceptible *P*. *aeruginosa* (26/48)	44.8%		
Chen et al. (2012)	ICU	MRSA (25/75)	33%	0.01	[48]
		MSSA (8/43)	18.6%		
Fu et al. (2015)	ICU	XDR *A*. *baumannii* (31/39)	79.5%	0.1	[49]
		Non-XDR *A*. *baumannii* (38/86)	44.2%		
Jia et al. (2015)	ICU	Linezolid non-susceptible Enterococci (3/44)	6.8%	0.521	[50]
		Linezolid-susceptible Enterococci (2/44)	4.5%		
		Un-infected Control patients (3/176)	1.7%		
Yao et al. (2015)	ICU	MRSA (12/57)	21%	0.002	[35]
		MSSA (9/116)	8%		
Gomez Rueda et al. (2014)	ICU	Carbapenem resistant *K*. *pneumoniae* (31/61)	50.8%	0.042	[36]
		Carbapenem-susceptible *K*. *pneumoniae* (20/61)	32.7%		
		Un-infected control patients (25/122)	20.4%		
Kumar et al. (2014)	ICU	Carbapenem-resistant *A*. *baumannii* (9/33)	27.3%	0.074	[37]
		Carbapenem-susceptible *A*. *baumannii* (3/32)	9.4%		
Nazer et al. (2015)	ICU	MDR-*A*. *baumannii* (118/161)	73.3%	0.015	[53]
		Non-MDR-*A*. *baumannii* (142/232)	61.2%		
Deris et al. (2011)	ICU	Imipenem-resistant -*A*. *baumannii* (6/15)	42.9%	0.201	[39]
		Imipenem-susceptible *A*. *baumannii* (9/41)	24.3%		
Inchai et al. (2015)	ICU	MDR-*A*. *baumannii* (10/72)	13.9%	0.001	[44]
		XDR- *A*. *baumannii* (88/220)	40%		
		PDR*-A*. *baumannii* (7/12)	58.3%		
Jamulitrat et al. (2009)	ICU	Imipenem-resistant-*A*. *baumannii* (35/67)	52.2%	0.001	[59]
		Imipenem-susceptible *A*. *baumannii* (26/131)	19.9%%		
Thatrimontrichai et al. (2016)	ICU	Carbapenem-resistant *A*. *baumannii* (10/63)	15.9%	0.01	[19]
		Carbapenem-susceptible *A*. *baumannii* (1/13)	7.7%		
		Un-infected control patients (0/25)	0%		
Topeli et al. (2000)	ICU	MRSA (15/46)	32.6%	0.02	[21]
		MSSA (7/55)	12.7%		

CR: Carbapenem-resistant; CS: Carbapenem susceptible; MBL: Metallo-beta-lactamase; IS: imipenem sensitive; IR: imipenem resistant; ICU: Intensive Care Unit; OSSA: Oxacillin-sensitive-*S*. *aureus;* ORSA: Oxacillin-resistant-*S*. *aureus*; PDR: Pan drug resistant; XDR: Extensive drug resistant

### Statistical analysis

#### Primary analyses

**Fig 3:**
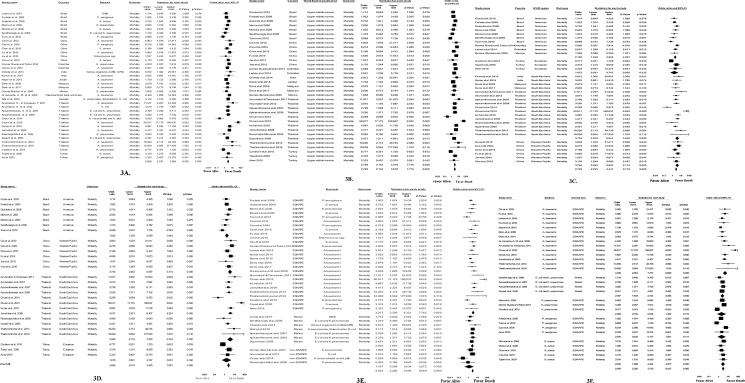
Forest plot of impact of ABR on mortality and sub-group analyses per World Bank classification, WHO regions, countries, group of bacteria and bacteria species. 3A. Forest plot of overall impact of antibiotic-resistance on mortality in included studies. 3B. Forest plot of impact of ABR on mortality analyzed per World Bank Classification. 3C. Forest plot of impact of ABR on mortality analyzed per WHO regions. 3D. Forest plot of impact of ABR on mortality analyzed per countries. 3E. Forest plot of impact of AMR on mortality analyzed per group of bacteria. 3F. Forest plot of impact of ABR on mortality analyzed per bacterial species.

Pooled estimates revealed 90% prevalence (95%CI, 2.852–3.557; p = 0.000) of mortality attributable to infections in developing countries with greater mortality associated with ABR at an odds ratio (OR) 2.828 (95%CI, 2.231–3.584; p = 0.000) (Fig 3A).

#### Subgroup analyses

The subgroup analyses were performed by World Bank classification, WHO region, country, group of bacteria and bacterial species. Fig 3B presents a forest plot of mortality due to AMR categorized per World Bank classification. The risk of mortality due to resistant bacteria was high in upper middle-income countries (OR 2.769, 95% CIs, 2.142–3.579; p = 0.000), with studies from lower-middle and low-income nations not being evaluated due to insufficient data.

Four out of the six WHO regions were included in the analysis, with three showing a high risk of mortality (Fig 3C). High statistical significance was observed in the Americas (OR 2.126, 95% CIs; 1.546–2.925; p = 0.000), South East Asia (OR 3.754, 95% CIs; 2.333–6.041; p = 0.000) and the Western Pacific (OR 3.746, 95% CIs; 2.463–5.697; p = 0.000) (Fig 3C). Results from Europe were not statistically significant and insufficient reports precluded analysis in Africa.

Subgroup analyses per country showed high statistical significance (OR 2.665, 95%CIs; 2.074–3.425, p = 0.000) (Fig 3D) in favor of mortality. Brazil, China and Thailand, had statistically significant risk of mortality with OR being 1.825 (95%CIs; 1.239–2.689; p = 0.002), 3.746 (95%CIs; 2.463–5.697; p = 0.000), 3.928 (95%CIs; 2.116–7.293; p = 0.000) respectively, in contrast to Turkey, which was not statistically significant (Fig 3D). In other countries, the number of reports was insufficient (less than three) to perform the meta-analysis.

Studies were categorized into three groups of bacteria namely ESKAPE, non-ESKAPE, and mixed (both ESKAPE and non-ESKAPE). The ESKAPE group was associated with the highest risk of mortality with a high statistical significance (OR 3.217; 95%CIs; 2.395–4.321; p = 0.001) (Fig 3E). Although, the non-ESKAPE group was not associated with the risk of mortality (OR 1.167; 95%CIs; 0.385–3.534; p = 0.000), when combined with ESKAPE within a study, it became statistically significant (OR 2.634; 95%CIs; 1.858–3.734; p = 0.000) (Fig 3E).

High risk of mortality due to antibiotic-resistant *A*. *baumannii* was observed with high statistical significance (OR 4.636; 95%CIs; 2.954–7.277; p = 0.000), followed by *S*. *aureus* (OR 2.842; 95%CIs; 1.868–4.324; p = 0.000). *P*. *aeruginosa* (OR 2.076; 95%CIs; 0.833–5.177; p = 0.117) and *K*. *pneumoniae* (OR 2.026; 95%CIs; 0.733–5.598; p = 0.173) were not significantly associated with mortality (Fig 3F).

## Discussion

AMR is a global public health threat that affects human health, particularly hospitalized patients, and has substantive health and financial consequences. This study analyzed the published literature on the clinical and economic implications of ABR in developing countries from 40 eligible studies. Antibiotic-resistant bacteria were associated with increased mortality (OR 2.8341, 95%CIs; 2.2180–3.6213; P = 0.000), consistent with several reports in both developed and developing countries [66–69]. The main ward involved was the ICU, possibly due to the heavy use of antibiotics and hence the selection pressure for ABR development and prevalence in these units [4, 23, 70, 71]). This concurred with studies from Mexico, Brazil, China, Thailand, France and Serbia, that reported high mortality due to antibiotic-resistant bacteria in ICUs [17, 49, 67, 71–73]. The study further showed that ABR research is neglected in developing countries with only one report from low-income (Senegal), two from lower-income (Palestine and Jordan), and 37 from upper-middle income nations (Table 1 and Fig 2). Developing countries are thus far behind high resource settings in the fight against AMR and that requiring considerable efforts to reduce its consequences [74]. Three WHO regions, i.e., the Americas, South East Asia and the Western Pacific region showed the highest risk of mortality associated with MRSA and *K*. *pneumoniae* resistant to third generation cephalosporins. Our results concurred with the 2014’s WHO report, which showed a significant increase of mortality due to antibiotic-resistant *K*. *pneumoniae* and *S*. *aureus* in hospitals particularly in ICU across WHO regions [2]. Resistance levels could be explained by the practices of self-medication and the purchase of antibiotics over-the-counter common in these settings. Policies and regulations promoting rational antibiotic use are also minimal or non-existent. Additionally, limitations in managing nosocomial infections, sub-optimal infection control measures, unsafe water, poor hygienic conditions, lack of knowledge and inadequately trained personnel might also be associated with the prevailing resistance in these regions. Comprehensive studies are needed to provide accurate and reliable data to inform decision-makers about the danger of ABR in developing countries and suggest a way forward for the alleviation of the resulting implications.

Resistant ESKAPE bacteria including carbapenem-resistant *A*. *baumannii*, MBL- producing *P*. *aeruginosa*, ESBL-producing *K*. *pneumoniae*, and MRSA represented the most common resistant bacteria associated with increased mortality. These bacteria were the main cause of morbidity and mortality in bloodstream infections in hospital settings, with a high statistical significance (OR 2.978, 95%CIs; 2.362–3.753; p = 0.000) (Fig 3F). This concurred with the WHO Global Antimicrobial Surveillance System (GLASS), which recognized *A*. *baumannii*, *K*. *pneumoniae*, and *S*. *aureus*, as priority pathogens in blood specimens and list them together with *P*. *aeruginosa* as priority antibiotic resistant-bacteria for research and development in 2017 [4, 5].

According to the meta-analysis, MDR-ESKAPE were associated with a greater risk of mortality than mono-drug (including imipenem, methicillin, and linezolid) resistant bacteria, with a high statistical significance (OR 2.846, 95% CIs; 1.744–4.643; p = 0.000; versus OR 2.301; 95%CIs; 1.718–3.082; p = 0.000; Table 3). Moreover, when comparing the mortality risk between resistant- and susceptible-ESKAPE pathogens (Table 3), results showed that carbapenem-resistant *A*. *baumannii* (CRAB) were associated with higher mortality risk than susceptible strains with a high statistical significance [2, 5]. The pooled estimate of mortality rate ranged from 15.9 to 91.7% (p = 0.001), consistent with a report from Taiwan, where a significant increase of mortality from 14% to 46% (p = 0.0001) was associated with carbapenem-resistant-*A*. *baumannii* implicated in HAIs during 2003–2008 [75].

Although the mortality attributable to ESKAPE pathogens is indisputable compared to non-ESKAPE pathogens, we observed that when these two groups infected patients concomitantly, they were associated with a long length of hospital stay (LOS) and a higher mortality. This concurred with studies from Senegal [55], Turkey [3] and China [35, 50] which have reported high LOS and death due to MDR-*A*. *baumannii*, ESBL-producing *Enterobacteriaceae* and MRSA, respectively.

Eight studies reported that ABR increased health care costs with resistant ESKAPE bacteria being the main causative agents associated with high hospital costs (Table 4). Four out of the eight revealed that length of stay had an impact on hospital costs. LOS was also a risk factor for acquisition of nosocomial infections, and thereby increased mortality. Overall, health-care costs in all studies for case and control groups were 8,107.375 USD versus 5,469.487 USD respectively. Two studies indicated health care costs >10 000 USD in Thailand and Colombia [19, 51] while one report showed cost ≥ 35 000 USD in Turkey [3]. In contrast, three studies reported overall hospital costs ≤ 1000 USD [55–57], with one below 250 USD in Senegal [55]. These differences are attributed to the diverse socio-economic characteristics of the countries concerned.

**Table 4:** Summary of data on health care costs associated with resistant infections.

Country	WHO Region	World Bank classification	Settings	Follow-up period	Overall Health care costs			References
					Case group	Control group	p-value	
Colombia	Americas (PAHO)	Upper Middle Income	Tertiary hospital	30 days	11 822 USD	7 178 USD	< 0.001	[51]
India	South East Asia (SEARO)	Upper middle income	Tertiary hospital	NR	1 478 USD	790 USD	< 0.001	[52]
Senegal	Africa (AFRO)	Low income	Hospital	NR	228 USD	122 USD	< 0.0001	[55]
Thailand	South East Asia (SEARO)	Upper middle income	University Hospital	34 days	935 USD	122 USD	< 0.05	[56]
Thailand	South East Asia (SEARO)	Upper middle income	University Hospital	43 days	615 USD	214 USD	< 0.05	[57]
Thailand	South East Asia (SEARO)	Upper middle income	University Hospital	NR	2731 USD	1 199 USD	< 0.001	[58]
Thailand	South East Asia (SEARO)	Upper middle income	University Hospital	NR	11 773 USD	7 797.9 USD	< 0.05	[19]
Turkey	Europe(EURO)	Upper middle income	University Hospital	28 days	35 277 USD	26 333 USD	< 0.282	[3]

**Fig 4:**
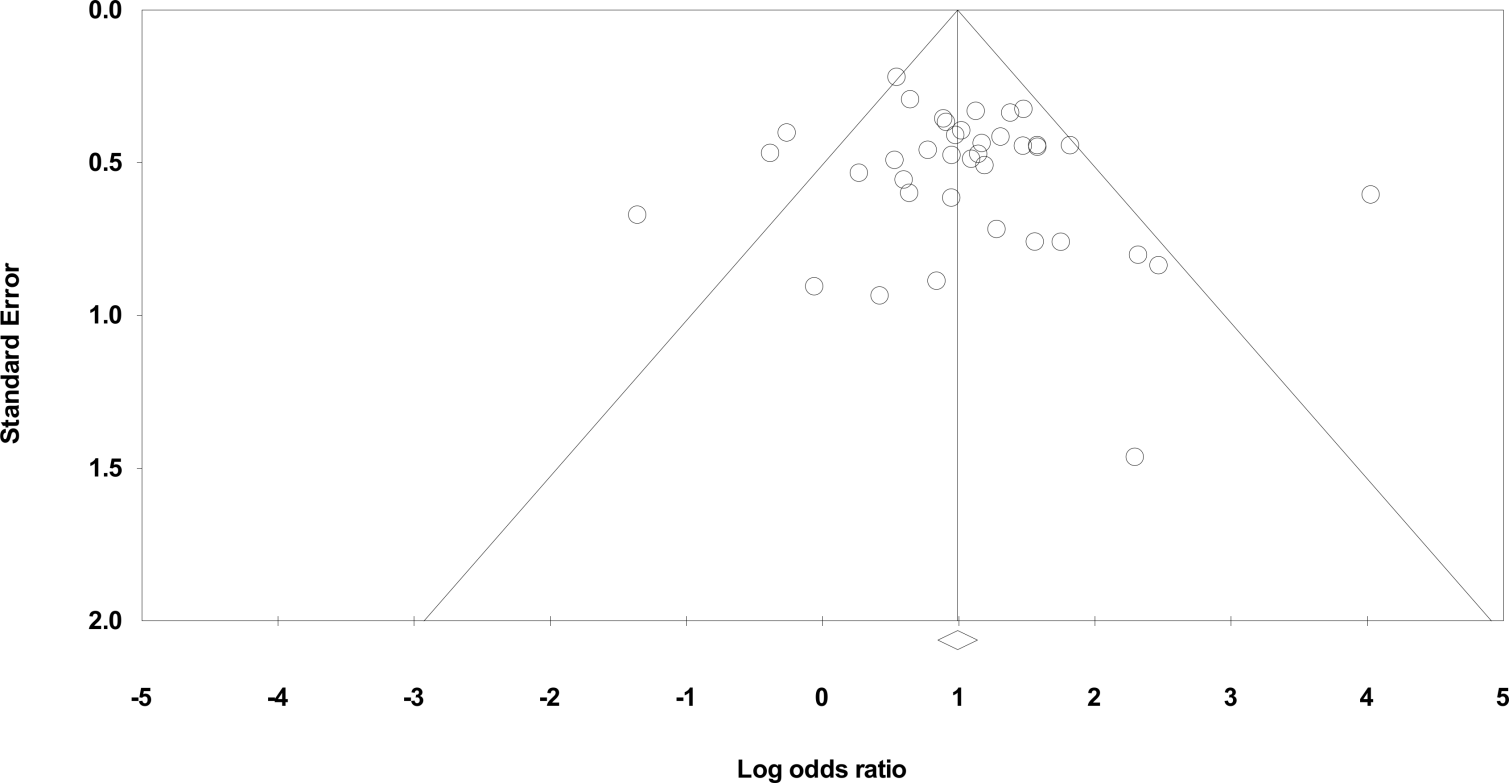
Funnel plot of standard error by log odds ratio.

In terms of the limitations of the study, several papers were not included in the meta-analysis because they did not provide sufficient information regarding clinical and/or economic impact of ABR in developing countries. We were unable to present the genomic characteristics of antibiotic-resistant bacteria due to the scarcity of data. In addition, we did not focus on antibiotic classes and resistance patterns due to the lack of standard methods for identification and interpretation in developing countries. Moderate heterogeneity (*I*^*2*^ *=* 58.88%, *p =* 0.000) was reported, which could be due to various external factors, such as different type of studies (retrospective, retrospective cohort, retrospective case-control, prospective cohort, prospective case-control, etc.), diverse populations (adult, children, neonates), infection prevention and control measures and antimicrobial stewardship practices. Moreover, minor publication bias was observed in the funnel plot (Fig 4) which could possibly be attributed to the lack of reports from lower-middle and low-income countries. We tried to limit the influence of heterogeneity and publication bias in our statistical analysis by using the random effects model that considers differences within and between studies, as well as by including articles in different languages (English and French).

## Conclusion and recommendations

The key findings of this study confirm that ABR, particularly antibiotic-resistant ESKAPE pathogens are associated with a high risk of mortality and greater economic costs. Developing countries need to optimize their management of communicable and non-communicable diseases, implement infection, prevention and control (IPC) measures, as well as antimicrobial stewardship programs (ASP) in both hospital and community settings to reduce morbidity, mortality and the costs associated with ABR. Furthermore, optimization of rational antibiotic use at regional and national levels, is essential to ensure a high quality and effective of therapeutic options [76]. Substantial and sustainable efforts to develop rapid diagnostics, new antibiotics and vaccines are required. An international platform for global real-time surveillance and monitoring of antimicrobial resistance could advance containment of this threat.

## Supporting information

S1 Table PRISMA checklist. (DOCX)

S2 Table Search strategy performed in PubMed and Web of Science. (DOCX)
