# Supplementary material for: Clinical and economic impact of antibiotic resistance in developing countries: A systematic review and meta-analysis
Source: PLoS One. 2017 Dec 21;12(12):e0189621. doi: 10.1371/journal.pone.0189621 (PMC5739407; doi:10.1371/journal.pone.0189621)
Supplement: S2 Table — (DOCX) [file pone.0189621.s002.docx]

**Clinical and Economic Impact of Antibiotic Resistance in Developing Countries: A Systematic Review and Meta-Analysis**

**Raspail Carrel Founou*, Luria Leslie Founou, Sabiha Yusuf Essack**

***Correspondence to:**

Raspail Carrel Founou

czangue@yahoo.fr

**Supplementary Table 2.** Search strategy performed in Pubmed and Web of Science

| ***Initial search terms*** | **Refining terms (AND/OR)** | **Restriction (NOT)** |
| --- | --- | --- |
| *“Antimicrobial* resistan*”*  *“Antibiotic resistan*”*  *“Drug* resistan*”*  *“Multi-drug resistan*”*  *“Multidrug resistan*”*  *“Multiple-drug resistan*”*  *“Multiple drug* resistan*”*  *Bacterial* resistan**  *Cross infection*  *Nosocomial*  *Hospital-acquired*  *Healthcare acquired*  *Hospital associated*  *Healthcare associated*  *Community acquired*  *Community-associated*  *Community*  *Hospital*  *Hospitals*  *Hospitalized*  *Intensive care*  *Critical care*  *Inpatients*  *Outpatients*  *Community patients* | Afghanistan, Albania, Algeria, American Samoa, Angola, Argentina, Armenia, Azerbaijan, Bangladesh, Belarus, Belize, Benin, Bhutan, Bolivia, Bosnia and Herzegovina, Botswana, Brazil, Bulgaria, Burkina Faso, Burundi, Cabo Verde, Cambodia, Cameroon, Central African Republic, Chad, China, Colombia, Comoros, Congo, Dem. Rep. Congo, Rep. Costa Rica, Cote d’Ivoire, Cuba, Djibouti, Dominica, Dominican Republic, Ecuador, Egypt, Arab Rep, El Salvador, Equatorial Guinea, Eritrea, Ethiopia, Fiji, Gabon, Gambia, The Georgia, Ghana, Grenada, Guatemala, Guinea, Guinea-Bissau, Guyana, Haïti  Honduras, India, Indonesia, Iran, Islamic Rep  Iraq, Jamaica, Jordan, Kazakhstan, Kenya, Kiribati, Korea Dem. People’s Rep, Kosovo, Kyrgyz, Republic Lao PDR, Lebanon, Lesotho, Liberia, Libya, Macedonia FYR, Malawi, Malaysia, Maldives, Mali, Marshall Islands, Mauritania, Mauritius, Mexico, Micronesia. Fed Sts, Moldova, Mongolia, Montenegro, Morocco, Mozambique, Myanmar, Namibia, Nepal, Nicaragua, Niger, Nigeria, Pakistan, Palau, Panama, Papua New Guinea, Paraguay, Peru, Philippines, Romania, Russian Federation, Rwanda, Samoa, Sao Tome and Pincipe, Senegal, Serbia, Sierra Leone, Solomon Islands, Somalia, South Africa, South Sudan, Sri Lanka, St Lucia, St Vincent and the Grenadines, Sudan, Suriname, Swaziland, Syrian Arab Republic, Tajikistan, Tanzania, Thailand, Timor-Leste, Togo, Tonga, Tunisia, Turkey, Turkmenistan, Tuvalu, Uganda, Ukraine, Uzbekistan, Vanuatu, Venezuela, RB Vietnam, West Bank and Gaza, Yemen, Rep. Zambia, Zimbabwe | *Review*  *Letter*  *Case reports*  *Meta-analysis*  *Editorial*  *Conference abstract*  *Meta-analysis*  *Correspondence*  *Comment*  *Year Restriction (2000)* |

Initial search terms refer to words used to filter out reports addressing antimicrobial resistance in the population of interest. Refining terms were then applied together with boolean operators (AND, OR) to select articles from the region of study. Truncation marks (*) specifies that diverse extensions were used.
